# Primary hepatic neuroendocrine carcinoma diagnosed by needle biopsy: a case report

**DOI:** 10.1186/s40792-021-01315-3

**Published:** 2021-11-02

**Authors:** Yusuke Seki, Hiroki Sakata, Toshimasa Uekusa, Hirokazu Momose, Satomi Yoneyama, Akio Hidemura, Yusuke Tajima, Hiroyuki Suzuki, Masahiro Ishimaru

**Affiliations:** 1 Department of Surgery, Kanto Rosai Hospital, 1-1 Kizukisumiyoshicho, Nakahara-ku, Kanagawa, 211-8510 Japan; 2 Department of Pathology, Kanto Rosai Hospital, 1-1 Kizukisumiyoshicho, Nakahara-ku, Kanagawa, 211-8510 Japan; 3 Department of Hepato-Biliary-Pancreatic Surgery, Kyorin University Hospital, 6-20-2 Shinkawa, Mitaka City, Tokyo 181-8611 Japan

**Keywords:** Primary hepatic neuroendocrine carcinoma, Chemotherapy, Surgical resection

## Abstract

**Background:**

Primary hepatic neuroendocrine carcinomas (NECs) are extremely rare. The rate of recurrence after resection is extremely high, and the prognosis is poor. It is debatable whether chemotherapy or surgical resection is the optimal initial treatment for primary hepatic NECs. Therefore, selecting an appropriate therapeutic approach for patients with primary hepatic NECs remains clinically challenging. We present a case of primary hepatic NEC in a patient who developed recurrence after undergoing surgical resection.

**Case presentation:**

A 78-year-old man with bone metastases of prostate cancer was referred to our department because of a solitary 66-mm tumor in the left lateral segment of the liver, which was detected on annual follow-up by computed tomography after prostate resection. A biopsy and preoperative diagnostic workup identified the lesion as a primary hepatic neuroendocrine carcinoma; therefore, left lateral segmentectomy was performed. Immunohistochemically, the tumor was positive for chromogranin A, synaptophysin, and CD 56, and the Ki-67 index was 40%. This neuroendocrine carcinoma was classified as a large cell type. Adjuvant chemotherapy with carboplatin + etoposide was initially administered a month after surgery. However, lymph node recurrence occurred 4 months after surgery, and the patient died of systemic metastases 15 months after surgical resection.

**Conclusions:**

Due to the lack of availability of abundant quantities of relevant, high-quality data, there is no standard therapy for primary hepatic NECs. Selecting the most appropriate treatment for patients depending on several factors, such as the stage and differentiation of a tumor and a patient’s performance status and clinical course, is consequently preferred. More cases need to be studied to establish the best treatment strategy for primary hepatic NEC.

## Background

Primary hepatic neuroendocrine carcinomas (NECs) are extremely rare, and radical surgical resection is considered the only therapeutic option for their treatment. However, the rate of recurrence and hematogenous or lymphogenous metastasis after surgical resection is extremely high, and the prognosis is poor. It is debatable whether the optimal initial treatment for primary hepatic NEC is chemotherapy or surgical resection, and selecting an appropriate therapeutic approach for such patients remains clinically challenging. Herein, we present a case of primary hepatic NEC in a patient who developed a recurrence after undergoing surgical resection.

## Case presentation

A 78-year-old man with a tumor in the liver detected during follow-up computed tomography (CT) for prostate cancer resected 11 years ago was referred to our hospital. He had undergone salvage radiation and antiandrogenic therapy for bone metastases from prostate cancer 2 years before the current visit to the hospital.

On presentation, his general condition was good. On palpation, the abdomen was soft and non-tender, and no shifting dullness was noted to suspect ascites. Laboratory test results did not reveal any liver dysfunction. The tests for hepatitis B virus surface antigen and antibodies for hepatitis C virus were negative. The levels of serum tumor markers alpha-fetoprotein (AFP, 2.8 IU/mL), carcinoembryonic antigen (CEA, 2.3 ng/mL), and cancer antigen 19-9 (CA 19-9, 9.2 ng/mL) were within the normal limits, whereas neuron-specific enolase (25.5 ng/mL) and pro-gastrin-releasing peptide (8050 pg/mL) levels were significantly high.

**Fig. 1:**
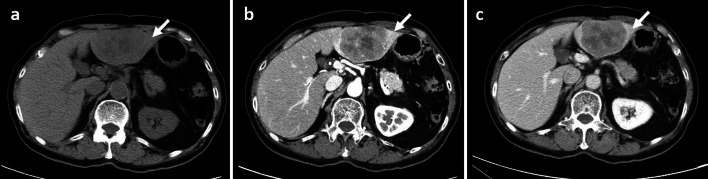
**a**–**c** Contrast-enhanced computed tomography (CECT): **a** plane; **b** arterial phase; and **c** portal venous phase. CECT showing a large well-defined and low-density mass with a diameter of 66 × 55 mm in the left lateral segment of the liver. The mass appears to have a lower density than the surrounding liver tissue on plane computed tomography images (**a**) and enhanced heterogeneously (**b**). No ascites or enlarged lymph nodes were detected

**Fig. 2:**
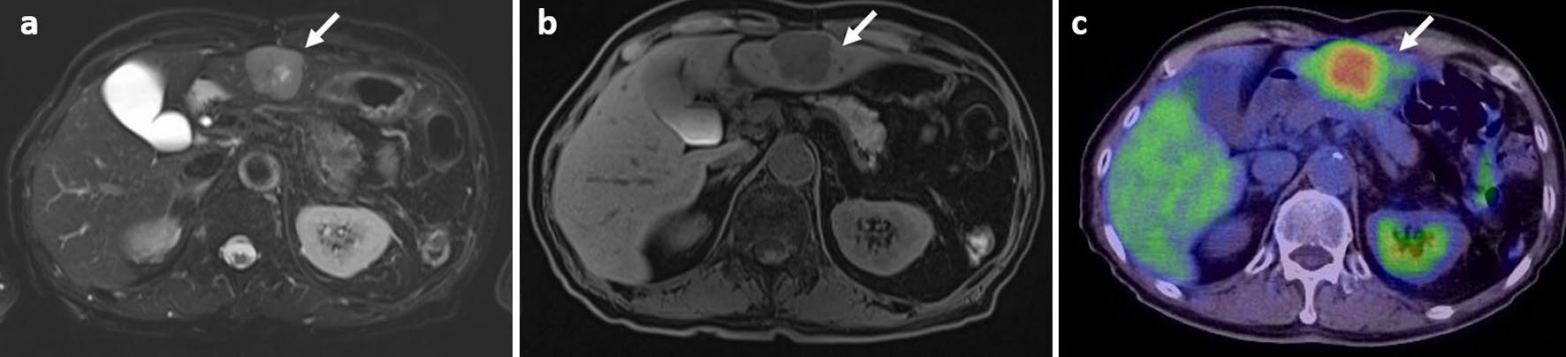
**a**–**c** Magnetic resonance imaging (MRI) and computed tomography: **a** fat-saturation T1-weighted image; **b** fat-saturation T2-weighted image; and **c** combined positron emission tomography and computed tomography (PET–CT). MRI showing a large mass in the left lateral segment of the liver, which is indicated by a low-intensity signal on T1-weighted images and a high-intensity signal on T2-weighted images and DWI. PET–CT showing that the maximum standardized uptake value of the tumor in the left lateral segment of the liver was 12.6

Contrast-enhanced CT showed a well-defined, large, low-density mass (66 × 55 mm in diameter) in the left lateral segment of the liver (Fig. 1). Magnetic resonance imaging (MRI) revealed a low-intensity mass on fat-saturation T1-weighted images and a high-intensity mass on fat-saturation T2-weighted images (Fig. 2). On combined positron emission tomography and CT (PET–CT), the maximum standardized uptake value of the tumor in the left lateral segment of the liver was considerably high (12.6) (Fig. 2).

To confirm the histological diagnosis of the liver tumor and distinguish between liver metastasis of prostate cancer and other primary malignancies, we performed a liver biopsy. Hematoxylin and eosin staining revealed round tumor cells with a high N:C ratio showing solid tumor nests. The nuclei of the tumor cells varied in size and shape. Tumor cells at the solid nest showed peripheral palisading. Mitotic activity was not observed in the field. Immunohistochemically, the tumor exhibited diffuse positivity for the expression of chromogranin A and synaptophysin and negativity for the expression of CD56. The Ki-67 index was 70%. The tumor was diagnosed histologically and immunohistochemically as a poorly differentiated NEC of uncertain origin. Preoperative examinations (upper and lower endoscopies, systemic CT, MRI, and PET) did not reveal a primary malignancy, except that found in the liver. Eventually, the tumor was clinically diagnosed as a primary hepatic NEC. At the cancer board in our hospital, we discussed and presented the patient with two options: surgical resection with adjuvant chemotherapy or chemotherapy for the initial treatment. The patient decided to undergo surgical resection because of anxiety regarding chemotherapy. Left lateral segmentectomy was performed. The postoperative course was uneventful, and the patient was discharged on postoperative day 9.

**Fig. 3:**
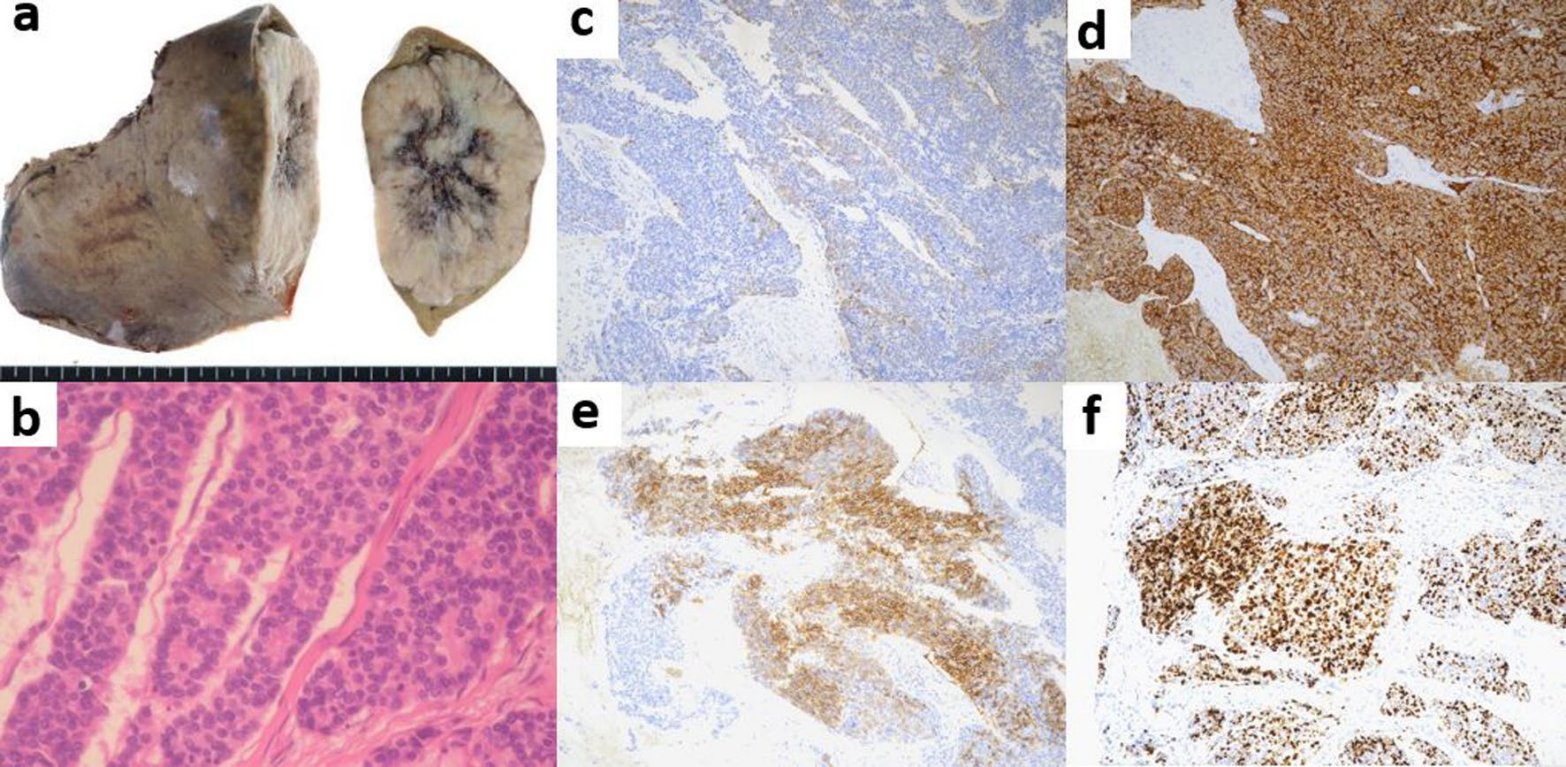
Macroscopic and histopathological findings of the resected specimen. **a** The resected specimen was a solid tumor with a diameter of 75 × 55 × 42 mm and with a clear border. The resection margins were tumor-free (R0 resection). The non-tumorous portion of the liver was normal. **b** Hematoxylin and eosin staining showed atypical cells with round hyperchromatic nuclei and scanty cytoplasm, forming large and small tumor nests. The tumor was arranged in rosette-like structures around the small blood vessels. Necrosis was identified at the center of the tumor nests, and high mitotic activity was observed in the 28/10 high-power field. **c–f** Immunohistochemical examination revealed that the tumor cells were diffusely positive for chromogranin A (**c**) and synaptophysin (**d**) and focally positive for CD 56 (**e**). The Ki-67 index was 40% (**f**)

Hematoxylin and eosin staining revealed atypical cells with round hyperchromatic nuclei and scanty cytoplasm, forming large and small tumor nests. The tumor was arranged in rosette-like structures around the small blood vessels. Necrosis was identified at the center of the tumor nests, and high mitotic activity was observed in a 28/10 high-power field (Fig. 3a, b). Immunohistochemically, the tumor exhibited diffuse positivity for the expression of chromogranin A and synaptophysin and focal positivity for the expression of CD56. The Ki-67 index was 40% (Fig. 3c–f). The lesion was classified as a large cell NEC.

Adjuvant chemotherapy with carboplatin + etoposide was initially administered a month after surgery. After the first course, he was admitted to our hospital for febrile neutropenia (Grade 3) and thrombocytopenia (Grade 4). Four months after surgery, lymph node recurrence behind the portal vein of the hepatoduodenal ligament occurred. We reinitiated the same regimen with the modified dose, and CT showed partial response under the response evaluation of Progression Disease by Response Evaluation Criteria in Solid Tumors after the second course. After the third course, we stopped chemotherapy because of anemia (Grade 3) and changes in the activities of daily living level. The patient selected best supportive care and chemotherapy was terminated. The patient died of systemic recurrence, including residual liver metastasis, 15 months after surgery.

## Discussion

The classification of neuroendocrine neoplasms (NENs) in the 2019 WHO Classification of Tumours of the Digestive System (5th edition) distinguishes well-differentiated neuroendocrine tumors (NETs), including previously designated carcinoid tumors when occurring in the GI tract, from poorly differentiated NECs based on genetic evidence, as well as clinical, epidemiological, histological, and prognostic differences [1]. Furthermore, NETs are graded as G1 (low grade), G2 (intermediate grade), and G3 (high grade), based on the assessment of proliferation activity, such as mitotic count and the Ki-67 proliferation index [1]. Nevertheless, all NECs are highly aggressive neoplasms and are generally subclassified into small cell NEC (SCNEC) and large cell NEC (LCNEC) [1]. Among all NENs, primary hepatic NETs and NECs are extremely rare, accounting for 0.3% of NETs and 0.28–0.46% of malignant liver tumors [2]. Additionally, primary hepatic NECs occur infrequently. Several previous studies have shown that primary hepatic NETs usually occur in middle-aged patients and have no sex predominance [3, 4]. The most common chief complaint of patients with NETs is abdominal pain [5]. Previous studies have reported that tumor markers, such as AFP, CEA, and CA 19-9, have no diagnostic value with respect to primary hepatic NEC [6].

The clinical diagnosis of primary hepatic NEC remains challenging because of its rarity and the lack of information about its characteristic appearance on images. On CT, primary hepatic NEC appears as a low-density mass with an enhanced margin, and the center of the mass is not enhanced due to necrosis [7]. MRI shows a low-intensity mass on fat-saturation T1-weighted images and a high-intensity area on fat-saturation T2-weighted images. Based on the abovementioned clinical-imaging findings, it is difficult to distinguish hepatic NECs from other hepatic carcinomas, such as hepatocellular carcinoma and cholangiocellular carcinoma. Consequently, pathological examination through the performance of a preoperative liver tumor biopsy is essential for diagnosis; however, surgical resection is often performed without preoperative liver biopsy.

When NEC of uncertain origin is diagnosed by liver tumor biopsy, it is extremely important to perform preoperative workup, including gastroscopy, colonoscopy, and PET–CT examinations, because neuroendocrine neoplasms of the liver are usually metastatic from other organs, such as the gastrointestinal tract and pancreas.

A final diagnosis of NECs is usually made based on pathological confirmation. On hematoxylin and eosin staining, NECs have a less nested architectural pattern, often growing in sheets. SCNEC has tightly packed fusiform nuclei with finely granular chromatin, whereas LCNEC has more rounded, markedly atypical nuclei and, sometimes, prominent nucleoli [1]. Immunohistochemical (IHC) staining of these tumors reveals immunoreactivity to general neuroendocrine markers, including chromogranin A and synaptophysin [1]. Although IHC markers effectively identify primary hepatic NENs, there is no specific IHC stain for hepatic NEC. IHC markers for NEC remain similar to those for common NENs, including chromogranin A (89.1%) and synaptophysin (48.9%), as previously reported by researchers [5].

There is no standard therapy for primary hepatic NEC. However, surgical resection is currently considered the most appropriate therapy for localized primary hepatic NENs. Undoubtedly, surgery is the only option for a cure and provides the most favorable outcome [8]. Elmer and Quartey reported that 84.5% of the 124 reported cases of primary hepatic NENs were treated with surgery with a mean disease-free interval of 33.6 months and 5-year survival of 75% [8]. However, among the primary hepatic NENs, primary hepatic NECs are extremely rare, and little is known about their clinical features and treatment outcomes [2, 9, 10]. Ishibe et al. reported that the 1-year survival rate for 20 reported cases of primary hepatic NECs was 23.5%, and the 5-year survival rate was 5.8%, indicating that the prognosis of primary hepatic NEC is extremely poor [11]. Park et al. reported that the median overall survival of 12 patients with primary hepatic NEC was 16.5 months (range 0.7–41.7 months), whereas the overall survival without recurrence of three patients who underwent surgical treatment was 17.7 months (range 15.2–36.9 months). Thus, surgical resection must be considered for curative intent [3]. Surgical resection of NEC is the most common treatment, particularly for localized primary hepatic NEC, whereas transarterial chemoembolization (TACE) is normally performed for advanced primary hepatic NECs that are poor candidates for resection [3, 12]. TACE can be used for cytoreduction of NETs because NETs are hypervascular and sensitive to ischemia [3]. Due to the lack of availability of substantial high-quality data, there is no standard therapy for primary hepatic NEC [2, 9, 10, 12]. Therefore, selecting an appropriate treatment depending on several factors such as tumor stage and differentiation and a patient’s performance status and clinical course is preferred [2, 9–12].

According to our research about primary hepatic neuroendocrine tumors, only 33 reports of surgery for primary hepatic NEC were published between 1980 and 2019 [3, 7, 13–27] (Table 1). The median age of the 34 patients, including our patient, was 56 years (range 8–78 years), and 21 patients (61%) were men. Eighteen of the 34 patients (52%) experienced recurrence (Table 1). Fourteen of the 18 patients (77%) developed recurrence within 6 months. We could not determine the risk factors for early recurrence in this cohort. The prognosis after recurrence was extremely poor. Twelve of the 18 patients developed recurrence in the remnant liver, and in some cases, the sites of recurrence were the hepatoduodenal ligament and lung.

**Table 1:** Summary of cases in which patients developed primary hepatic neuroendocrine carcinoma recurrence following surgical resection

First author	Year	age	sex	Tumor size (cm)	Solitary (yes/no)	Treatment before surgery	Recurrence (months)	Site of recurrence	Treatment after recurrence	Survival (months)
Hsueh	1983	8	F	17	Yes	Chemotherapy	None	None	None	4
Zancotani	1996	56	M	5	Yes	None	3	Liver	None	5
Pilichowska	1999	57	M	8.2	Yes	None	ND	Liver	None	16
Ishida	2003	72	M	3	Yes	None	ND	ND	None	ND
Garcia	2006	50	M	5	Yes	None	4	Liver	TACE, Chemotherapy	16
Yang	2009	65	M	7.5	Yes	None	3	Liver	None	12
		56	M	ND	No	None	None	None	ND	36.9
		68	F	ND	No	None	None	None	ND	18
		51	F	ND	No	Chemotherapy	6.2	ND	ND	15.2
Akahoshi	2010	64	M	1.5	Yes	None	None	None	ND	3
Huang	2010	51	M	ND	No	TACE	48	Liver	ND	107
		34	M	ND	No	TACE	None	None	ND	98
		52	F	ND	No	None	5	Liver	ND	47
		59	M	ND	No	None	None	None	None	34
		54	M	ND	No	None	None	None	None	24
		43	M	ND	No	None	None	None	None	15
		50	F	ND	No	None	5	Liver	ND	14
		37	M	ND	No	None	1	Liver	PEIT	13
		58	F	ND	No	None	39	ND	ND	148
		56	F	ND	No	None	5	Liver	ND	33
		50	M	ND	No	None	3	Liver	ND	12
Chan Hyuk	2012	51	F	ND	No	Chemotherapy	None	ND	ND	36.9
		68	F	ND	No	None	None	ND	ND	18
		51	F	ND	No	Chemotherapy	6.2	ND	ND	15.2
Shinkawa	2013	73	M	5	Yes	None	4	Bone, LN	ND	6
Kim	2013	67	F	9	Yes	None	None	None	None	3
Kano	2014	73	M	3	Yes	None	6	Liver	Chemotherapy	10
Sotiropoul	2014	19	F	27	Yes	None	None	None	None	24
Aboelene	2014	51	M	20	Yes	None	None	None	None	6
Choi	2016	72	M	2.2	Yes	None	6	Liver	Chemotherapy	4
Nakatake	2017	62	M	5	Yes	None	3	LN	Chemotherapy	24
Harada	2017	69	F	3.5	Yes	None	1	Liver, Lung	Chemotherapy	19
Xin	2020	64	F	1.8	Yes	None	ND	ND	None	5
Yusuke	2021	78	M	6.6	Yes	None	4	LN	Chemotherapy	4

*TACE* transarterial chemoembolization, *F* female, *M* male, *ND* not described, *LN* lymph node

In our case, although adjuvant chemotherapy with carboplatin + etoposide was initially administered a month after surgery, lymph node recurrence behind the portal vein of the hepatoduodenal ligament was found 4 months after surgery. The patient died of systemic recurrence, including residual liver metastasis, 15 months after surgery.

Whether surgical resection, in this case, contributed to the prognosis is unclear. Most patients with primary hepatic NEC undergoing surgical resection tend to develop recurrence within a relatively short period after surgery. Therefore, in the National Comprehensive Cancer Network Guidelines version 2 in 2018, several treatment options have been advocated for resectable NECs, including resection and adjuvant chemotherapy with or without radiation, neoadjuvant chemotherapy with or without radiation and resection, and chemotherapy alone [28]. Due to the aggressive course and poor prognosis, it is recommended that adjuvant chemotherapy be administered as soon as possible after surgery.

As for chemotherapy, the use of platinum-based drugs (cisplatin or carboplatin) and etoposide resulted in good outcomes, which led to their use as the standard regimen [29]. In our case, adjuvant chemotherapy with carboplatin + etoposide was initiated a month after surgery [7]. However, following major surgical resection, a period of at least 1–2 months is necessary before the initiation of chemotherapy. It remains unclear whether the proliferation of tumors is accelerated during this period. Therefore, chemotherapy has the potential to be considered as the initial treatment of choice, even for surgically resectable carcinomas [29]. Therefore, it is extremely important to evaluate tumor proliferation according to immunohistochemical findings and the need for surgical resection [30, 31]. Several patients with primary hepatic NEC still require a combined approach with not only surgical resection but also transcatheter arterial embolization, chemotherapy, and ultrasound-guided radiofrequency ablation [7, 28]. Variable treatment modalities should be decided based on each patient’s performance status and desire for a type of treatment. Selecting appropriate therapeutic approaches remains clinically challenging. Further studies are necessary to establish a standard approach.

## Conclusion

Due to the lack of availability of abundant amounts of high-quality data, there is no standard therapy for primary hepatic NECs. Selecting the most appropriate treatment for patients depending on several factors, such as the stage and differentiation of a tumor and a patient’s performance status and clinical course, is preferred.

## Data Availability

The patient data of this case report will not be shared to ensure patient confidentiality.

## Abbreviations

CT: Computed tomography
MRI: Magnetic resonance imaging
NEN: Neuroendocrine neoplasms
NET: Neuroendocrine tumor
NEC: Neuroendocrine carcinoma
SCNEC: Small cell NEC
LCNEC: Large cell NEC
IHC: Immunohistochemical
TACE: Transarterial chemoembolization
PET: Positron emission tomography
PET–CT: Combined positron emission tomography and computed tomography
AFP: Alpha-fetoprotein
CEA: Carcinoembryonic antigen
CA 19-9: Cancer antigen 19-9
